# Altered Amplitude of Low-Frequency Fluctuations in Inactive Patients with Nonneuropsychiatric Systemic Lupus Erythematosus

**DOI:** 10.1155/2019/9408612

**Published:** 2019-11-25

**Authors:** Yang Yu, Liheng Chen, Qiaohong Wang, Lingzhen Hu, Qiuping Ding, Xize Jia, Xuyan Yang

**Affiliations:** ^1^Department of Psychiatry, Second Affiliated Hospital, College of Medicine, Zhejiang University, 88 Jiefang Rd, Hangzhou 310009, China; ^2^Department of Rheumatology, Second Affiliated Hospital, College of Medicine, Zhejiang University, 88 Jiefang Rd, Hangzhou 310009, China; ^3^Center for Brain Imaging Science and Technology, Key Laboratory for Biomedical Engineering of Ministry of Education, College of Biomedical Engineering and Instrumental Science, Zhejiang University, 38 Zheda Rd, Hangzhou 310027, China; ^4^Center for Cognition and Brain Disorders, Institutes of Psychological Sciences, Hangzhou Normal University, Zhejiang Key Laboratory for Research in Assessment of Cognitive Impairments, No. 2318 Yuhangtang Rd, Hangzhou 311121, China

## Abstract

**Objective:**

This study is aimed at investigating the characteristics of the spontaneous brain activity in inactive patients with nonneuropsychiatric systemic lupus erythematosus (non-NPSLE).

**Methods:**

Thirty-one female inactive patients with non-NPSLE and twenty healthy controls were examined by resting-state functional magnetic resonance imaging (RS-fMRI). Three amplitude methods including amplitude of low-frequency fluctuations (ALFF), fractional amplitude of low-frequency fluctuations (fALFF), and percent amplitude of fluctuation (PerAF) (with and without standardization) were applied to evaluate the spontaneous brain activity. The correlation was performed between low-frequency oscillations and clinical and neuropsychological factors in inactive patients with non-NPSLE.

**Results:**

Compared to healthy controls, patients with non-NPSLE showed increased standardized ALFF (mALFF) in the left inferior temporal gyrus and left putamen, decreased PerAF in the right postcentral gyrus and bilateral precentral gyrus, and increased standardized PerAF (mPerAF) in the left putamen and decreased mPerAF in the right postcentral gyrus and bilateral precentral gyrus. By standardized fALFF (mfALFF), no significant brain regions were found between the two groups. Correlation analysis revealed significantly positive correlations between glucocorticoid dose and PerAF in the right precentral gyrus and mPerAF in the left putamen, and Complement 3 (C3) and mPerAF in the right postcentral gyrus. There was a significant negative correlation between C3 and mALFF in the left putamen.

**Conclusion:**

Abnormal low-frequency oscillations in multiple brain regions were found in inactive patients with non-NPSLE, indicating that the alteration of mALFF, PerAF, and mPerAF in specific brain regions might be an imaging biomarker of brain dysfunction in inactive patients with non-NPSLE.

## 1. Introduction

Neuropsychiatric manifestations (NP) are common in patients with systemic lupus erythematosus (SLE) [1–3]. The symptoms of neuropsychiatric SLE may vary, such as mild mood disorder to psychosis or status epilepticus that accounts for up to 19% of deaths in patients with SLE [4–6]. Moreover, neuropsychological studies have found that SLE patients with nonneuropsychiatric systemic lupus erythematosus (non-NPSLE) can be affected with cognitive abnormalities [7]. The review of 22 published studies found that cognitive impairment was seen in 72% to 75% of non-NPSLE patients, which may be associated with lower health-related quality of life indices [8]. Thus, it is critical to understand the underlying neural substrate of cognitive impairments in non-NPSLE patients.

As a noninvasive method for investigating the physiological brain activity, resting-state functional magnetic resonance imaging (RS-fMRI) has been broadly applied for neuropsychiatric diseases [9–11]. Recently, RS-fMRI researches indicated reduced connectivity within the default mode network (DMN), the central executive network (CEN), and in-between the DMN and CEN in SLE [12]. Abnormal cortical thickness in the left lingual gyrus was found to be associated with increased resting-state functional connectivity of the left posterior cingulate cortex in non-NPSLE patients [13]. By both RS-fMRI and task-based fMRI, the abnormal functional connectivity of frontal-parietal was found to be associated with diseased activity in SLE patients [14]. Besides the abnormalities of functional connectivity, there is also evidence of brain activity alteration in SLE patients. Using the regional homogeneity (ReHo) approach, which reflected intraregional synchronization, decreased regional activity in areas of the default mode network and cerebellum was revealed in SLE patients [15]. Importantly, the spontaneous low-frequency (typically 0.01-0.1 Hz) oscillations (LFOs) of the human brain are thought to reflect the changes of spontaneous neuronal and physiological activities to a certain extent [16]. Decreased coupling between ALFF and functional connectivity density (FCD) in bilateral hippocampus-parahippocampus was found in non-NPSLE patients in combination with ALFF and FCD together, and correlated with C3, C4, and Montreal Cognitive Assessment [17]. Previous studies of RS-fMRI mainly focused on active patients with non-NPSLE. However, the brain activity in inactive patients with non-NPSLE remains elusive.

Recently, ALFF and fraction amplitude low-frequency fluctuations (fALFF) have been widely applied to the RS-fMRI studies of different types of disease, such as amnestic mild cognitive impairment, depression, schizophrenia, and dyspepsia [9, 10, 18, 19]. ALFF, as a reliable approach to monitor spontaneous neuronal fluctuations, can reflect cerebral physiological states [20–22]. Though ALFF is applied to investigate the brain neural function, it could be easily influenced by the respiratory and cardiac signals [22]. To effectively inhibit nonspecific signal components of RS-fMRI, fALFF is applied to measure range of low frequency (0.01-0.08 Hz) divided by the entire frequency range. Compared with ALFF, fALFF can provide better one-sample *t*-test pattern in the default mode network [23]. However, some articles showed that ALFF tended to have higher reliability than fALFF in the test–retest reliability of amplitude measures [22]. Percent amplitude of fluctuation (PerAF) has been proved to be more reliable and promising in investigating the abnormal BOLD signal in RS-fMRI [24, 25]. Zhao et al. [26] found that PerAF has the best reliability among amplitude of low-frequency fluctuation (ALFF), regional homogeneity (ReHo), and degree centrality (DC). Because the scale of BOLD signal can affect the ALFF value, all the previously published ALFF papers used standardized ALFF for group-level statistical analysis. PerAF could provide both original amplitude map and standardized amplitude map (mPerAF) [24, 25]. Both PerAF and mPerAF are meaningful, so we used PerAF and mPerAF [24, 25].

In the present study, mALFF, mfALFF, PerAF, and mPerAF were used to explore the local spontaneous brain activity of SLE patients. Considering the situation that brain activities may be affected by the disease activity, only inactive patients with non-NPSLE were included in the present study. The correlations between low-frequency oscillations in significant brain regions and clinical and neuropsychological factors were explored in the patient group.

## 2. Methods

### 2.1. Participants

In this study, thirty-one SLE patients who fulfilled the American College of Rheumatology (ACR) diagnostic criteria of SLE [27] were recruited at the Department of Rheumatology of Second Affiliated Hospital of Zhejiang University. All patients had no history of neuropsychiatric symptoms classified according to ACR criteria [28]. The SLE disease activity index (SLEDAI) was used to assess the disease activity by an independent physician [29]. The inclusion criteria for all patients are as follows: (1) female, right-handed, and age ranging from 15 to 45 years; (2) inactive SLE at least 24 weeks; (3) no organ damage; and (4) all non-NPSLE patients were in an inactive condition with SLEDAI < 5 and had no organ damage for more than 6 months. Twenty female controls demographically matched with patients in terms of age, sex, and years of education were recruited from local community. The exclusion criteria for patients and healthy controls are as follows: (1) left-handed; (2) brain damage, such as head trauma and a clear history of stroke; (3) nervous system diseases, such as Parkinson's disease; (3) drug and alcohol abuse; (4) pregnancy; (5) major depression (Beck Depression Inventory (BDI‐II) > 13) [30]; (6) any physical condition, such as hypertension and diabetes; and (7) positive antiphospholipid antibody (aPL).

This study protocol was approved by the Ethics Committee of the Second Affiliated Hospital of Zhejiang University. All participants signed a written informed consent before the study.

### 2.2. Clinical and Laboratory Data and Psychometric Tests

After obtaining the written informed consent, we collected blood samples of patients which included Complement 3 (C3), Complement 4 (C4), anti-dsDNA, and antiphospholipid antibody while scanning was performed. In half an hour after scanning, all the participants finished the Fatigue Severity Scale (FSS) [31], the Beck Depression Inventory (BDI-II), and Mini-Mental State Examination (MMSE). The higher value of FSS and Beck Depression Inventory represent worse symptoms and the higher value of MMSE represents better cognitive function. Current medications such as glucocorticoids (GC), hydroxychloroquine, cyclophosphamide, and mycophenolate mofetil (MMF) were obtained from the health records and medical files.

### 2.3. Data Acquisition

A total of 240 time points (8 min) were collected on a 3T MR scanner (SIEMENS MAGNETOM Prisma) for each subject, equipped with a 64-channel phased array head coil at 3T MRI center, Zhejiang University. The resting-state fMRI was acquired using an echoplanar imaging (EPI) sequence with the following parameters: (repetition time (TR) = 2000 ms, echo time (TE) = 30 ms, 78° flip angle, 3.4 mm slice thickness, 220 mm field-of-view, voxel size = 3.4 × 3.4 × 3.4 mm^3^). Moreover, the anatomical T1-weighted images were recorded by magnetization prepared rapid gradient echo: (repetition time (TR) = 2300 ms, echo time (TE) = 2.26 ms, 8° flip angle, 1 mm slice thickness, 256 mm field-of-view, voxel size = 1 × 1 × 1 mm^3^). All subjects were asked to lie quietly in the scanner and close their eyes in the process of data collection.

### 2.4. Data Preprocessing

Image data preprocessing was carried out using RESTplus V1.2 (http://www.restfmri.net) [24] and SPM12 (https://www.fil.ion.ucl.ac.uk/spm/). The preprocessing steps included: (1) The first 10 volumes were discarded to achieve steady-state magnetization and allow participants to adapt to fMRI noise. (2) Slice timing correction. (3) Realign. We excluded the participants of head motion exceeding 2 mm or 2° and then participants whose head motion (Mean FD Jenkinson et al. [32]) were greater than 2^∗^SD above the mean motion of the participants (threshold: 0.1284) were excluded considering the standard of head motion. Finally, three patients and three controls were excluded. We compared the head motions of the two groups using mean FD Jenkinson. The results showed that there was no significant difference between two groups in head motion (*p* = 0.869). (4) The T1-weighted images were coregistered with the average functional images and then segmented into the white matter (WM), gray matter (GM), and cerebrospinal fluid (CSF). (5) The functional images were spatially normalized by the file ‘y_‘imagename'. nii' into the Montreal Neurological Institute (MNI) space, and then resampled to 3 mm isotropic voxel size. (6) Spatial smoothing with a Gaussian kernel of 6 mm full-width at half-maximum (FWHM). (7) Removing the linear trend of the time series. (8) Regressing out nuisance variables, including Friston-24 head motion parameters [33], the cerebrospinal flow signals, and white matter signals. The mean value of the time series of each voxel was added back in this step.

### 2.5. RS-fMRI Measure Calculations

ALFF and fALFF analyses were performed using the RESTplus V1.2. After preprocessing, the time series of each voxel was transformed to frequency domain by fast Fourier transform (FFT), and the power spectrum was obtained. The square root was calculated at each frequency of the power spectrum. The average square root across 0.01-0.08 Hz was taken as ALFF of each voxel. The ratio of the sum of amplitude within the 0.01-0.08 Hz to that of the whole frequency band was calculated as fALFF [23]. For standardization, the ALFF value of each voxel was divided by the global mean ALFF within the brain mask and the fALFF value of each voxel was divided by the global mean fALFF within the brain mask.

PerAF is the percentage of resting-state BOLD fluctuation relative to the mean signal intensity of a given time series [25]. PerAF was calculated after preprocessing and bandpass (0.01-0.08 Hz) filtering. PerAF of each voxel was divided by the global mean PerAF, which lead to obtaining the mPerAF map.

### 2.6. Statistical Processes

Two-sample *t*-test was performed in the demographic data of both groups in SPSS (version 24.0, Armonk NY). All tests of demographic were two-tailed and *p* < 0.05 was considered significant. mALFF, mfALFF, PerAF, and mPerAF were analyzed by an independent two-sample *t*-test in the DPABI V3.1 [34]. Age, FD Jenkinson, and years of education were regressed in the two-sample *t*-test to avoid their influence. Multiple comparisons were performed by 3dClusterSim using AFNI 18.0.27. The FWHM were estimated by each residual map of statistical analysis (mALFF = [8.1, 8.2, 7.9], mfALFF = [7.6, 7.7, 6.8], PerAF = [8.7, 8.9, 8.5], and mPerAF = [7.1, 7.2, 6.8]). For mALFF, a voxel level of *p* < 0.005 and a contiguity threshold of 43 contiguous voxels were used as criteria for statistical significance corresponding to a corrected two-tailed *p* < 0.05. For mfALFF, a voxel level of *p* < 0.005 and a contiguity threshold of 36 voxels were used as criteria for statistical significance corresponding to a corrected two-tailed *p* < 0.05. For PerAF, a voxel level of *p* < 0.005 and a contiguity threshold of 52 contiguous voxels were used as criteria for statistical significance corresponding to a corrected two-tailed *p* < 0.05. For mPerAF, a voxel level of *p* < 0.005 and a contiguity threshold of 33 voxels were used as criteria for statistical significance corresponding to a corrected two-tailed *p* < 0.05. We saved the cluster with a significant group difference and then calculated the regional average value of each cluster. Pearson's correlation was performed separately for each variable to investigate the relationship between the clinical and neuropsychological factors of SLE patients (C3, C4, disease duration, GC doses, SLEDAI, FSS, and BECK scale) and the values of ALFF, fALFF, PerAF, and mPerAF in significant brain regions.

## 3. Results

### 3.1. Demographics and Clinical Data

Demographics and clinical data of patients with non-NPSLE and controls were displayed (Table 1). No significant differences in age (*p* = 0.405) and education level (*p* = 0.052) were found between patients with non-NPSLE and controls. All non-NPSLE patients were in an inactive condition with SLEDAI < 5 and had no organ damage for more than 6 months. There was a significant difference of FSS (*p* < 0.001) between non-NPSLE and controls. However, no significant differences of BECK and MMSE between patients and controls (both *p* > 0.05) were found.

### 3.2. Group Differences in mALFF and mfALFF

**Figure 1:**
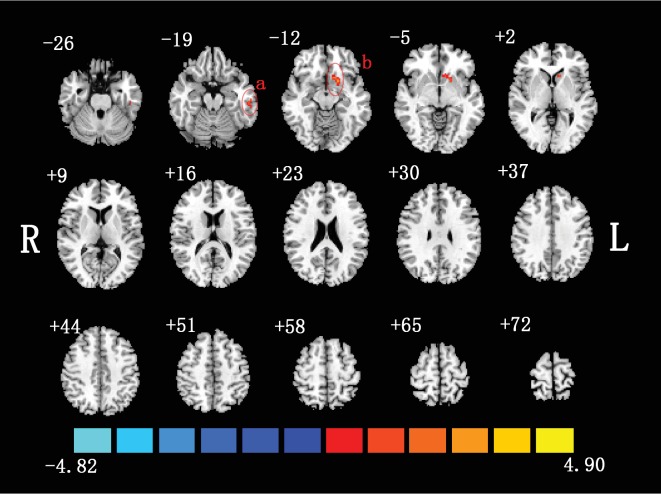
Two-sample *t*-test was performed between non-NPSLE patients and healthy controls (*p* < 0.05, corrected). Warm colors exhibited increased mALFF in non-NPSLE patients compared to healthy controls, and blue colors exhibited the opposite (a: left inferior temporal gyrus; b: left putamen; R: right hemisphere; L: left hemisphere). This figure was created in Slice Viewer [67].

The spatial distributions of mALFF in non-NPSLE patients and controls were shown (Figure 1). Compared to healthy controls, inactive patients with non-NPSLE showed increased ALFF in the left inferior temporal gyrus (ITG.L) and left putamen (PUT.L). These brain regions were summarized (Table 2). No significant brain region was found between patients and healthy controls by mfALFF.

### 3.3. Group Differences in PerAF

**Figure 2:**
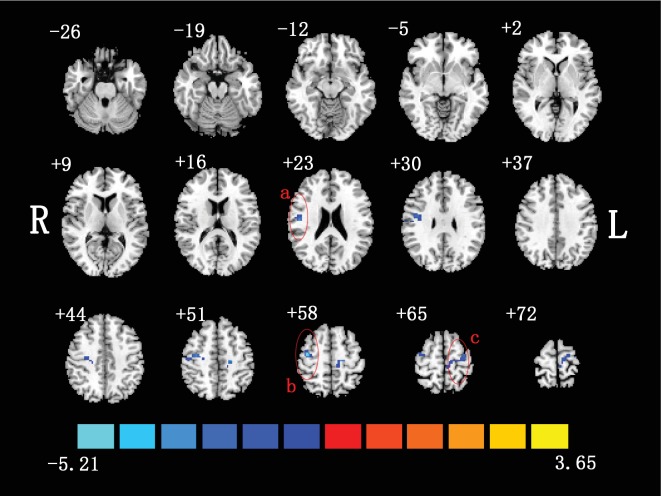
Two-sample *t*-test was performed between non-NPSLE patients and healthy controls (*p* < 0.05, corrected). Warm colors exhibited increased PerAF in non-NPSLE patients compared to healthy controls, and blue colors exhibited the opposite (a: right postcentral gyrus; b: right precentral gyrus; c: left precentral gyrus; R: right hemisphere; L: left hemisphere). This figure was created in Slice Viewer [67].

The PerAF differences between the inactive patients with non-NPSLE and the healthy controls were shown (Figure 2). Compared to healthy controls, patients showed decreased PerAF in the right postcentral gyrus (PoCG.R) and bilateral precentral gyrus (PreCG). These brain regions were summarized (Table 2).

### 3.4. Group Differences in mPerAF

**Figure 3:**
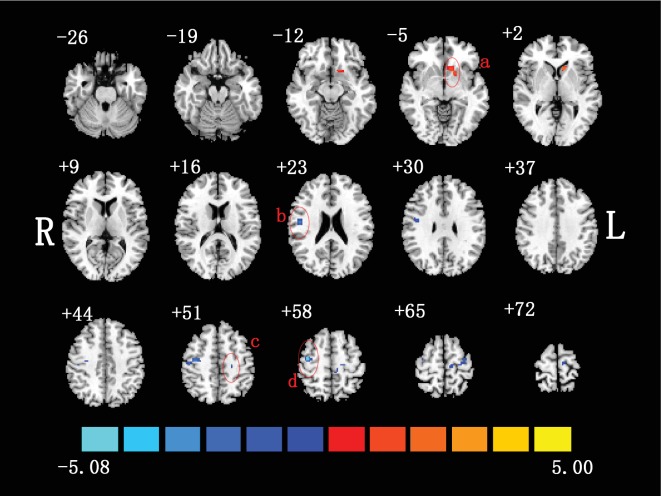
Two-sample *t*-test was performed between non-NPSLE patients and healthy controls (*p* < 0.05, corrected). Warm colors exhibited increased mPerAF in non-NPSLE patients compared to healthy controls, and blue colors exhibited the opposite (a: left putamen; b: right postcentral gyrus; c: left precentral gyrus; d: right precentral gyrus; R: right hemisphere; L: left hemisphere). The Figure 3 was created in Slice Viewer [67].

Compared to healthy controls, patients showed increased mPerAF value in PUT.L as well as decreased mPerAF value in PoCG.R and bilateral PreCG (Figure 3**)**. The brain regions, coordinates, and other summary information were shown in Table 2.

### 3.5. Pearson Correlations between Low-Frequency Oscillations and Clinical and Neuropsychological Factors

**Figure 4:**
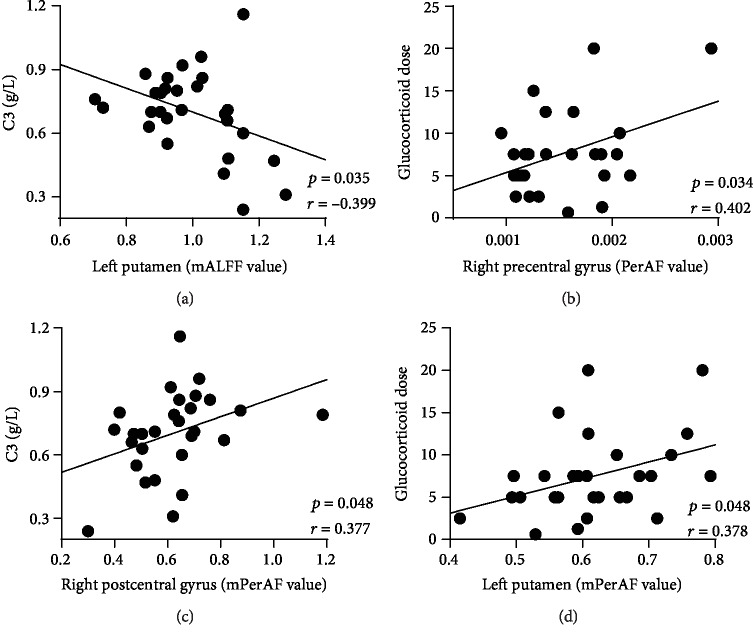
The mALFF of the left putamen was negatively correlated with C3 (a) (*r* = −0.399, *p* = 0.035). Correlation analyses showed significantly positive correlation between the PerAF in the right precentral gyrus with glucocorticoid dose (b) (*r* = 0.402, *p* = 0.034), the mPerAF in the right postcentral gyrus and C3 (c) (*r* = 0.377, *p* = 0.048), and the mPerAF in the left putamen and glucocorticoid dose (d) (*r* = 0.378, *p* = 0.048).

The correlations between the mALFF, PerAF, and mPerAF of significant brain regions and clinical factors (disease duration, GC doses, C3, C4, and SLEDAI) and neuropsychological factors (Beck, FSS, and MMSE) were performed. Pearson correlation analyses showed significantly positive correlation between the glucocorticoid dose and PerAF in the right precentral gyrus (*r* = 0.402, *p* = 0.034) and mPerAF in the left putamen (*r* = 0.378, *p* = 0.048), C3 and the mPerAF in right postcentral gyrus (*r* = 0.377, *p* = 0.048). Moreover, a significant negative correlation between C3 and the mALFF in the left putamen (*r* = −0.399, *p* = 0.035) was found (Figure 4). There was no significant correlation between mALFF, PerAF, or mPerAF values and SLEDAI score, disease duration, C4, psychometric scale FSS, BECK, and MMSE.

## 4. Discussion

In this present study, we utilized mALFF, mfALFF, PerAF, and mPerAF to investigate the spontaneous neural activity of inactive patients with non-NPSLE. Our results demonstrated abnormal brain functional regions including mALFF in the left inferior temporal gyrus (ITG.L) and left putamen (PUT.L), as well as the PerAF in the right postcentral gyrus (PoCG.R) and bilateral precentral gyrus (PreCG). Moreover, the patients exhibited abnormal mPerAF in PUT.L, PoCG.R, and bilateral PreCG compared to healthy controls. These findings may provide evidence of the dysfunctional brain patterns even in inactive patient with non-NPSLE.

Abnormal resting-state spontaneous brain activity in patients with non-NPSLE was found in previous studies [12–15, 17]. Zhang et al. has found the presence of abnormal mALFF in the inferior temporal gyrus (ITG) in active patients with non-NPSLE. Our study also presented the same results, which suggested that the abnormalities of spontaneous brain activity are present not only in active patients but also in inactive patients with non-NPSLE. ITG is considered an important brain area for the default mode network (DMN), which is associated with spontaneous cognition, monitoring of the external environment, and self-inspection [35–39]. ITG plays an important role in the processing of visual stimulus and is also involved in language fluency [36, 40–42]. Interestingly, previous studies showed patients with SLE frequently had cognitive dysfunction such as abnormal semantic fluency which resulted in negative impingement on social function [43–46]. In the present study, we performed MMSE that includes orientation, memory, attention, language fluency, apraxia, and alexia [47, 48]. MMSE is the most common tool to screen severe cognitive dysfunction and dementia, but its utility in the detection of mild cognitive dysfunction in SLE patients is limited [49]. In the present study, the difference of MMSE was unremarkable (*p* = 0.224) between the two groups, but our patients showed a lower score of MMSE. Hence, the abnormal mALFF in ITG and the lower score of MMSE in inactive patients with non-NPSLE suggested that the cognitive function may be affected.

In the study, we found the abnormality of both mALFF and mPerAF in the left putamen. The putamen, as a part of the striatum, receives axons from almost all parts of cortex [50]. It is well known that the functional interaction between dopaminergic neurotransmission and glutamate neurotransmission plays an important role in the integration of striatum [51]. By magnetic resonance imaging, neuroimaging studies provide important new insights into the structure and function of the putamen [52, 53]. There were animal studies which disclosed the existence of impaired dopamine catabolism and degenerating axon terminals in the brain of a lupus mouse [54, 55]. Maybe patients with non-NPSLE also have abnormal dopamine catabolism. Therefore, these findings provided support for the importance of the putamen in the SLE patients; further studies are needed for the underlying mechanism of the abnormal brain activity in lupus patients.

Furthermore, we found decreased PerAF and mPerAF in the postcentral gyrus and bilateral precentral gyrus in inactive patients with non-NPSLE. Using the task-based fMRI, a previous study found differences in the Wisconsin Card Sort Test between new onset SLE patients and healthy controls and reported the abnormal activation of the postcentral gyrus and precentral gyrus during feedback evaluation and response selection [56]. The postcentral gyrus is the primary somatosensory cortex located in the parietal lobe. The damage of the postcentral gyrus may lead to somatosensory impairment, mainly in tactile localization and postural sensitivity [57, 58]. The precentral gyrus is a key component of sensory and motor movement. Bernardo et al. [59] revealed that SLE patients with memory impairment showed reduced cortical thickness in the precentral gyrus when compared to the SLE patients without memory deficits. Previous diffusion tensor imaging study demonstrated lower fractional anisotropy in the precentral gyrus in SLE patients [60]. Sensorimotor network (SMN), including the postcentral gyrus and precentral gyrus, is one of the earliest resting brain network proposed in the fMRI study [16]. Nystedt et al. [12] found increased functional connectivity in the sensory motor network between SLE patients and healthy controls. The abnormality of the postcentral gyrus and precentral gyrus suggested that inactive patients with non-NPSLE may suffer from somatosensory impairment.

We also found that the mALFF of the putamen was correlated with serum C3; the PerAF in the precentral gyrus was correlated with glucocorticoid dose. Moreover, positive correlation between the right postcentral gyrus and C3, left putamen, and glucocorticoid dose were found. As a central molecule in the immune system, C3 is involved in the pathogenesis of SLE [61]. These correlations indicated that the abnormality of ALFF in the postcentral gyrus and putamen may be induced by C3-mediated immune inflammation. These results suggest that the postcentral gyrus and putamen may be specific targets of brain dysfunction in inactive patients with non-NPSLE, and C3 could be a biomarker to monitor their functions.

Interestingly, all of our patients are in a stable condition for at least 6 months and used a low-sustained glucocorticoid dose (7.48 ± 4.88 mg). We found significant positive correlations between two brain regions (the left putamen and right precentral gyrus) and glucocorticoid dose, suggesting the low-sustained dose of glucocorticoid could influence the brain activity of the left putamen and right precentral gyrus. These findings indicated that in clinical, inactive patients with non-NPSLE should use as fewer glucocorticoid as possible in order to avoid brain function damage caused by glucocorticoid.

In the present study, compared to healthy controls, the inactive patients with non-NPSLE showed significantly higher score of fatigue. The prevalence of fatigue ranges from 76% to 100% in active or inactive SLE patients [62–64], so we thought fatigue is a common symptom of systemic lupus erythematosus. Previous studies found that patients with SLE had significantly higher scores of FSS compared to healthy controls, which is consistent with our study [12, 64–66]. We will carry out relevant researches in the future.

In addition, we found that the different brain regions between the two groups using mPerAF had some overlaps with those using mALFF; the different brain regions between the two groups using mPerAF had some overlaps with those using PerAF. mPerAF is more sensitive than mALFF, which is consistent with previous studies [25, 26]. Although fALFF may be more sensitive and specific to brain activity compared with ALFF [23], our study failed to find significant brain regions using fALFF measures. It may be weaker in test–retest reliability of amplitude measures [23].

Our study had some potential limitations. First, although the conditions of all patients were inactive, these patients need to take a quite small dose of prednisone and/or hydroxychloroquine, which may be a confounder on BOLD signals. However, it was a common limitation in functional MRI studies. Second, we did not do other cognitive tests besides MMSE. In further studies, we may perform neuropsychiatric battery to identify patients with cognitive dysfunction.

## 5. Conclusion

In conclusion, this study showed the alteration of mALFF, PerAF, and mPerAF in several brain regions of inactive patients with non-NPSLE. The resting-state brain function alteration in the right postcentral gyrus, left putamen, left inferior temporal gyrus, and bilateral precentral gyrus may affect a variety of cognitive abnormalities in inactive patients with non-NPSLE. mALFF, PerAF, and mPerAF in specific brain regions might be imaging biomarkers for preclinical monitoring brain dysfunction in inactive patients with non-NPSLE.

## Figures and Tables

**Table 1:** Clinical and laboratory findings in inactive patients with non-NPSLE and health controls.

Demographics	Non-NPSLE (*n* = 28)	HC (*n* = 17)	*p* value
Age (years)	35.54 ± 7.38	33.53 ± 8.35	0.405
Education (years)	12.21 ± 3.61	14.35 ± 3.24	0.052
Disease duration (years)	7.71 ± 5.58	N/A	N/A
SLEDAI	1.25 ± 1.17	N/A	N/A
Antiphospholipid antibodies	0	N/A	N/A
Treatments		—	—
Glucocorticoid (mg)	7.48 ± 4.88	N/A	N/A
Hydroxychloroquine	20(71.4%)	N/A	N/A
Mycophenolate mofetil	2(7.1%)	N/A	N/A
Cyclophosphamide	2(7.1%)	N/A	N/A
C3 (g/L)	0.70 ± 0.20	N/A	N/A
C4 (mg/L)	119.68 ± 65.23	N/A	N/A
MMSE	28.86 ± 1.35	29.29 ± 0.69	0.224
FSS	39.57 ± 9.74	26.82 ± 10.98	<0.001
BECK	7.11 ± 4.17	6.35 ± 3.95	0.552

Abbreviations: HC = health controls; SLEDAI = systemic lupus erythematosus disease activity index; FSS = Fatigue Severity Scale; BECK = Beck Depression Inventory II (BDI-II); MMSE = Mini-Mental State Examination; N/A - not applicable. Statistical analysis was two-tailed and *p* < 0.05 was considered significant.

**Table 2:** Brain regions showing mALFF, PerAF, and mPerAF differences between groups.

Brain regions	BA	Cluster size (no. voxels)	Peak MNI coordinates			*t* value
			*X*	*Y*	*Z*	
Increased mALFF in SLE patients						
Left PUT	48	60	-18	12	-9	4.19
Left ITG	20	44	-57	-27	-21	4.50
Decreased PerAF in SLE patients						
Right PoCG	43	66	54	-9	21	-4.45
Right PreCG	6	97	39	-12	57	-5.21
Left PreCG	48	111	-36	-12	69	-4.88
Increased mPerAF in SLE patients						
Left PUT	25	60	-15	15	-9	4.20
Decreased mPerAF in SLE patients						
Right PoCG	48	39	54	-9	21	-4.69
Right PreCG	6	61	39	-12	54	-5.08
Left PreCG	6	56	-21	-21	54	-4.61

MNI: Montreal Neurological Institute; BA: Brodmann area; PoCG: postcentral gyrus; PUT: putamen; ITG: inferior temporal gyrus; PreCG: precentral gyrus.

## Data Availability

All the files (mALFF, mfALFF, PerAF, mPerAF) used to support the findings of this study have been deposited in http://www.restfmri.net/SLE/.
